# Correlation of Fractional Flow Reserve with non-invasive tests for the detection of ischaemia due to intermediate coronary artery stenosis

**DOI:** 10.1186/1532-429X-13-S1-P85

**Published:** 2011-02-02

**Authors:** Timothy A Fairbairn, Adam Mather, John Greenwood, Sven Plein

**Affiliations:** 1 University of Leeds. Leeds, UK

## Objectives

To compare ischaemia assessment by Fractional flow reserve (FFR) with non-invasive testing in patients with intermediate coronary artery stenosis.

## Background

FFR was initially validated against SPECT, Dobutamine Stress Echo and Exercise Testing [1]. It is now frequently used to determine the management of intermediate coronary artery stenosis. A cut-off value of 0.75 is used in clinical practice to guide revascularisation supported by long-term outcome data [2], but a ‘grey zone’ of 0.75-0.8 with uncertain clinical significance exists [3]. Advances in non-invasive imaging tests (gated SPECT and CMR) warrant a re-evaluation of FFR at intermediate stenosis severity against non-invasive imaging.

## Methods

Patients due for investigation of presumed cardiac chest pain were recruited and underwent SPECT (Discovery, GE Healthcare), perfusion-CMR (1.5T, Intera, Phillips) and coronary angiography. Any vessel that was angiographically determined as intermediate severity (40-70%) was assessed by QCA and pressure wire-derived FFR (RADI medical systems, Uppsala, Sweden).

## Results

In 23 study patients (age 57±8, 78% male), 33 FFR measurements were performed (LAD 64%, Cx 18%, RCA 12%, LMS 6%). FFR was classified negative (>0.80) in n=20. Perfusion-CMR detected ischaemia in 3 vessels (2 with positive FFR and one with 'grey' FFR). SPECT also detected ischaemia in 3 vessels (2 negative FFR and one positive FFR), (Table 1). Coronary stenosis by QCA and FFR correlated poorly (r= -0.35, p=0.054) . Chi-squared analysis of FFR severity found no significant association between FFR positivity and perfusion-CMR (p=0.078) or SPECT (p=0.34).

**Table 1:** Cross tabulation of Fractional Flow Research grading (negative ≥0.8, grey 0.75-0.79 and positive <0.75) and the qualitative result of (A) Perfusion cardiac magnetic resonance (CMR) and (B) SPECT.

A		CMR		Total
		Non ischaemic	Ischaemic	
**FFR**	**Negative**	20	0	20
	**Grey**	7	2	9
	**Positive**	3	1	4
**Total**		30	3	33

**B**		**SPECT**		
		**Non ischaemic**	**Ischaemic**	**Total**

**FFR**	**Negative**	18	2	20
	**Grey**	9	0	9
	**Positive**	3	1	4
**Total**		30	3	33

## Conclusion

Non-invasive imaging does not correlate well with FFR measurements in intermediate coronary lesions. Perfusion-CMR whilst not significantly discriminating between the groups had no false negatives and may thus be the more useful additional test to determine the significance of ‘grey’ lesions on FFR.
